# Digital Lesions in Dogs: A Statistical Breed Analysis of 2912 Cases

**DOI:** 10.3390/vetsci8070136

**Published:** 2021-07-17

**Authors:** Julia Maria Grassinger, Andreas Floren, Tobias Müller, Argiñe Cerezo-Echevarria, Christoph Beitzinger, David Conrad, Katrin Törner, Marlies Staudacher, Heike Aupperle-Lellbach

**Affiliations:** 1 Laboklin GmbH & Co. KG, 97688 Bad Kissingen, Germany; cerezo@laboklin.com (A.C.-E.); beitzinger@laboklin.com (C.B.); conrad@laboklin.com (D.C.); toerner@laboklin.com (K.T.); aupperle@laboklin.com (H.A.-L.); 2 Institut für Tierökologie und Tropenbiologie, Universität Würzburg, 97070 Würzburg, Germany; floren@biozentrum.uni-wuerzburg.de; 3 Institut für Bioinformatik, Universität Würzburg, 97070 Würzburg, Germany; Tobias.Mueller@uni-wuerzburg.de; 4 AniCura Aachen, Dres. Staudacher, 52078 Aachen, Germany; m.staudacher@tgz-aachen.de

**Keywords:** canine, subungual, toe, tumor, inflammation, breed predisposition

## Abstract

Breed predispositions to canine digital neoplasms are well known. However, there is currently no statistical analysis identifying the least affected breeds. To this end, 2912 canine amputated digits submitted from 2014–2019 to the Laboklin GmbH & Co. KG for routine diagnostics were statistically analyzed. The study population consisted of 155 different breeds (most common: 634 Mongrels, 411 Schnauzers, 197 Labrador Retrievers, 93 Golden Retrievers). Non-neoplastic processes were present in 1246 (43%), tumor-like lesions in 138 (5%), and neoplasms in 1528 cases (52%). Benign tumors (n = 335) were characterized by 217 subungual keratoacanthomas, 36 histiocytomas, 35 plasmacytomas, 16 papillomas, 12 melanocytomas, 9 sebaceous gland tumors, 6 lipomas, and 4 bone tumors. Malignant neoplasms (n = 1193) included 758 squamous cell carcinomas (SCC), 196 malignant melanomas (MM), 76 soft tissue sarcomas, 52 mast cell tumors, 37 non-specified sarcomas, 29 anaplastic neoplasms, 24 carcinomas, 20 bone tumors, and 1 histiocytic sarcoma. Predisposed breeds for SCC included the Schnauzer (log OR = 2.61), Briard (log OR = 1.78), Rottweiler (log OR = 1.54), Poodle (log OR = 1.40), and Dachshund (log OR = 1.30). Jack Russell Terriers (log OR = −2.95) were significantly less affected by SCC than Mongrels. Acral MM were significantly more frequent in Rottweilers (log OR = 1.88) and Labrador Retrievers (log OR = 1.09). In contrast, Dachshunds (log OR = −2.17), Jack Russell Terriers (log OR = −1.88), and Rhodesian Ridgebacks (log OR = −1.88) were rarely affected. This contrasted with the well-known predisposition of Dachshunds and Rhodesian Ridgebacks to oral and cutaneous melanocytic neoplasms. Further studies are needed to explain the underlying reasons for breed predisposition or “resistance” to the development of specific acral tumors and/or other sites.

## 1. Introduction

Regardless of the underlying cause, dogs with digital lesions have well-known clinical signs, such as lameness, digital masses, ulcerations, and breaking or splitting of the toe nails [1,2]. Frequently, in order to diagnose and treat this condition, digital amputation and subsequent histologic evaluation is performed [1]. Of all of the canine digital conditions, inflammatory lesions account for 19–31.1%, while malignant neoplastic processes account for 53.5–81% [1,3,4]. Digital tumor-like lesions (mostly adnexal dysplasia and polyps) occur in 5.7 [4] to 6.4% [1] of cases. Digital benign neoplasms include subungual keratoacanthoma (22.4%), sebaceous adenoma (7.5%), histiocytoma (4.4%), keratoma (4.4%), plasmacytoma (3.0%), and trichoblastoma (3.0%) [1] as well as hemangioma (16%) and lipoma (8%) [3].

Subungual squamous cell carcinoma (SCC) is the most common (47.4%) diagnosed malignant digital tumor in dogs [1], usually occurring in dogs with a mean age of 10 years [5]. According to Grüntzig et al. [6] females have a lower risk of developing SCC than males. A breed predisposition has been described for Schnauzers, Rottweilers, standard Poodles, black Labrador Retrievers [6,7,8], Briards, and Beaucerons [5].

The second most frequent canine digital malignant neoplasia is the melanoma [3], a neoplasia originating from melanocytes [9]. This tumor has a reported prevalence of 16.2–17.3% [1,3,4] as well as an aggressive behavior [9]. Interestingly enough, there is no consensus regarding the initiating factors for this neoplasia in dogs [10]. Additionally, Schnauzers, Irish Setters [2], Golden Retrievers [10], Rottweilers, Poodles, and Labrador Retrievers [11] are high-risk breeds regarding the development of acral melanoma. Regardless of breed, black-coated dogs seem to be overrepresented [11].

Wobeser et al. [1] also described other canine digital malignant tumors, including soft tissue sarcoma (13%), mast cell tumors (8.7%), osteosarcoma (3%), round cell sarcoma (1.7%), and adenocarcinoma (1.3%). This correlates with data provided by Marino et al. [3] who identified a 10.5% prevalence of soft tissue sarcoma and a 1.3% prevalence of osteosarcoma.

Although these studies report some breed predispositions, the sample size is relatively small (Wobeser et al. [1]: *n* = 404, Gruber-Beckmann et al. [4]: *n* = 380, Marino et al. [3]: *n* = 117). On the other hand, recognizing tumor-resistant breeds may significantly contribute to a deeper understanding of oncogenesis. Individualized therapy and breeding decisions are based, among other factors, on the underlying genetic principles of tumors.

Therefore, the objectives of this study were (1) the evaluation of the neoplastic, non-neoplastic, and tumor-like lesions in the submitted surgically amputated canine digits on a larger scale (*n* = 2912) and (2) the statistical analyses of signalment data to identify breed predispositions as well as statistically less affected breeds.

## 2. Materials and Methods

This retrospective study reviewed 2912 canine amputated digits and claws out of 162,360 pathological submissions from the Laboklin GmbH & Co. KG, Bad Kissingen, Germany submitted by European veterinarians (2219 cases from Germany and 694 samples from 23 other European countries) during the years 2014–2019. Inclusion criteria were a preliminary report of the breed and a clear histopathological diagnosis.

### 2.1. Histopathology

All digital samples were fixed in 10% phosphate-buffered formalin, trimmed according to Kamstock et al. [12], and decalcified in a mixture of ≥10–<20% hydrochloric acid (HCl) and formaldehyde (≥3%–<5%) (Osteomoll^®^ rapid decalcifier solution for histology, Merck, Darmstadt, Germany) over a period of 24–72 h. Representative sample sites were processed for routine histopathological examination according to standard procedures. Sections were stained with hematoxylin and eosin (HE). For histological analysis and pictures, the selected slides were scanned using Aperio ImageScope (Leica, Wetzlar, Germany) and histologically evaluated by trained veterinary pathologists during routine diagnostics. The diagnosis of the tumor-like lesions and tumors were made following World Health Organization (WHO) classifications [13,14,15]. The data sets of the cases were extracted into an excel table. The data were standardized in terms of format and nomenclature for statistical analyses. The diagnoses were grouped into (1) non-neoplastic, (2) tumor-like lesions, and (3a) benign or (3b) malignant neoplasms.

### 2.2. Statistics

Statistical significance analyses of all of the included amputated digits were performed using R version 4.0.2 (R Foundation, Vienna, Austria) and IBM SPSS statistics (version 26, IBM, Armonk, NY, US). Using univariate logistic regression, the occurrence of canine digital tumors was modelled. The breed was added to this model. Mongrels were the reference level for the variable breed. Only breeds with more than 50 dogs were included within the statistical analyses. We applied Firth-type logistic regression with intercept-correction (FLIC) to obtain unbiased average predicted probabilities as implemented in the r package “logistf” [16]. Results of the regression models were visualized by using the package “sjPlot” [17]. All given significant values were corrected by multiple tests, and the *p*-values were adjusted for multiple testing based on the Benjamini–Hochberg correction [18]. Comparisons between the groups (non-neoplastic, tumor-like, neoplastic lesions) were performed using the Kruskal–Wallis test. *p*-values < 0.05 were considered statistically significant.

## 3. Results

**Figure 1:**
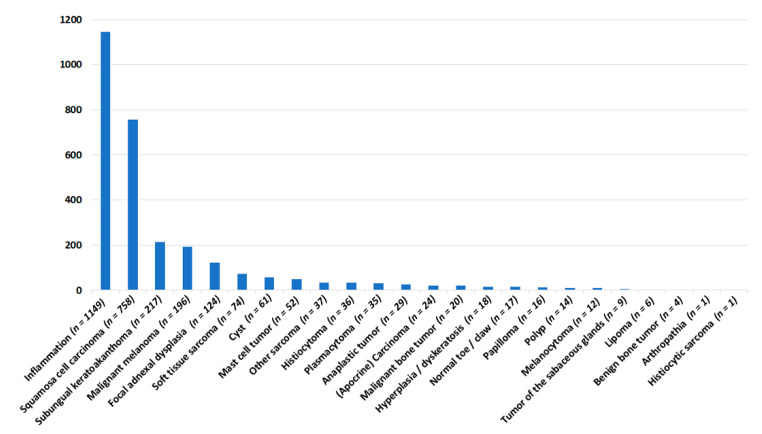
Histopathological main diagnoses in the digital samples of 2912 dogs submitted to Laboklin GmbH & Co. KG during the years 2014–2019.

In total, there were 27 different histopathological diagnoses on the toes and claws of 2912 dogs. The diagnoses are summarized as main diagnoses in Figure 1. They were grouped into (1) non-neoplastic, (2) tumor-like lesions, and (3a) benign or (3b) malignant neoplasms.

Non-neoplastic processes were present in 1246 cases (group 1, 43%), tumor-like lesions in 138 samples (group 2, 5%), and neoplasms in 1528 samples (group 3, 52%; 3a benign neoplasms, 22%; 3b malignant neoplasms, 78%). The study population consisted of 155 different breeds. The most common breeds included: 634 Mongrels, 411 Schnauzers (giant: *n* = 228, standard *n* = 183), 197 Labrador Retrievers, and 93 Golden Retrievers. The dogs had a median age of 9 years old (0.1–17 years), with males being overrepresented (intact: *n* = 1014, castrated: *n* = 531) compared to females (intact: *n* = 572, spayed: *n* = 582). The age was unknown in 295 cases, and the sex was unknown in 213 cases.

**Figure 2:**
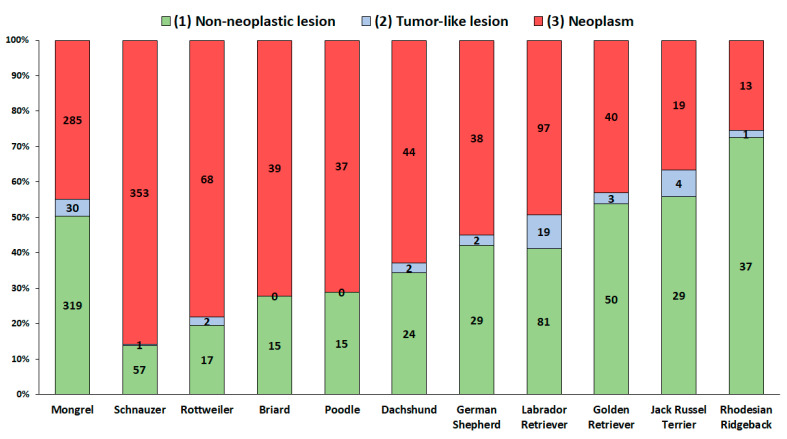
Distribution of non-neoplastic lesions, tumor-like lesions, and neoplasms in digital samples of the most common breeds (more than 50 dogs per breed).

Half of the digital samples from the Mongrels were diagnosed with tumors. Over 80% of the digital samples from Schnauzers and Rottweilers were neoplastic, in contrast to the Rhodesian Ridgebacks, of which only 30% had neoplastic changes (Figure 2).

**Figure 3:**
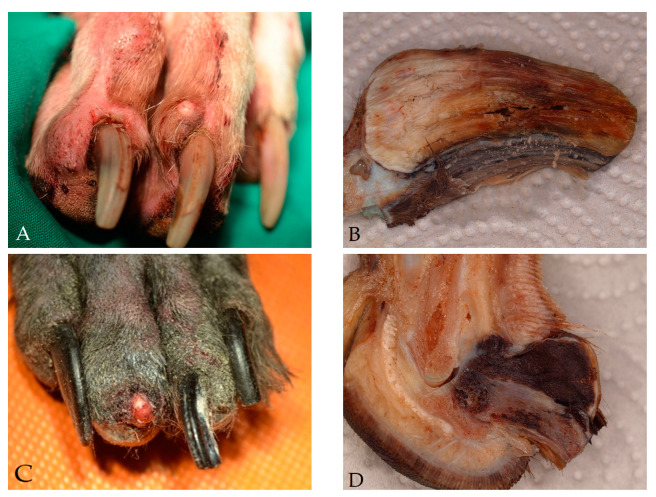

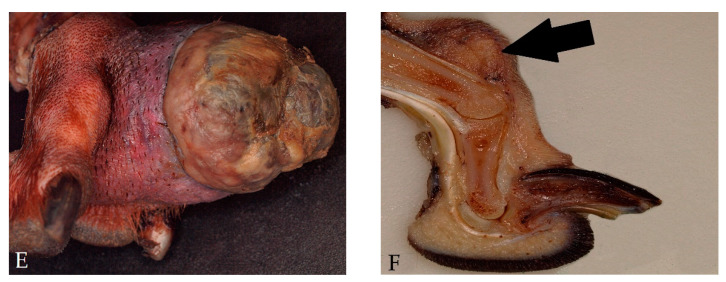
Gross pictures of various pathological lesions in canine toes. ((**A**,**C**) preoperative clinical pictures; (**B**,**D**,**F**) longitudinal cut, formalin fixed material; (**E**) formalin fixed amputated digits.) (**A**) Severe pyogranulomatous dermatitis with hair fragments and osteomyelitis of the nail beds of a 5- year-old Great Dane; (**B**) subungual keratoacanthoma of a 13-year-old Cavalier King Charles Spaniel; (**C**) squamous cell carcinoma (diameter 0.9 cm) of a Schnauzer with claw loss and osteolysis; (**D**) acral malignant melanoma (diameter 1.6 cm) of an 11-year-old Labrador Retriever; (**E**) ulcerated neurofibroma (diameter 3.0 cm) between two toes of a 13-year-old Mongrel; (**F**) Cutaneous mast cell tumor (arrow) in a 6-year-old German Shepherd dog.

**Figure 4:**
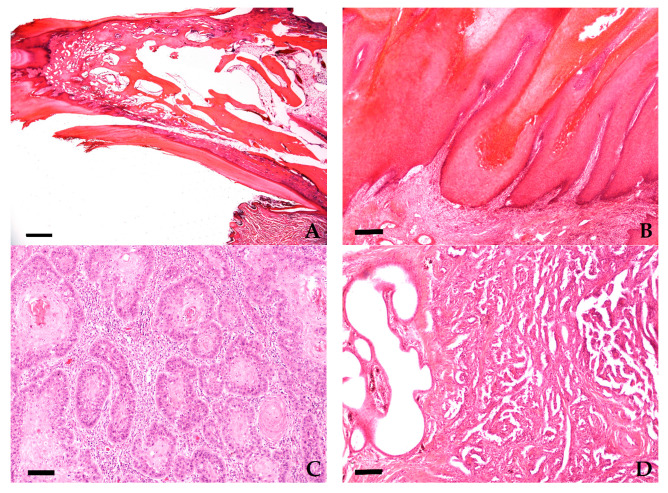

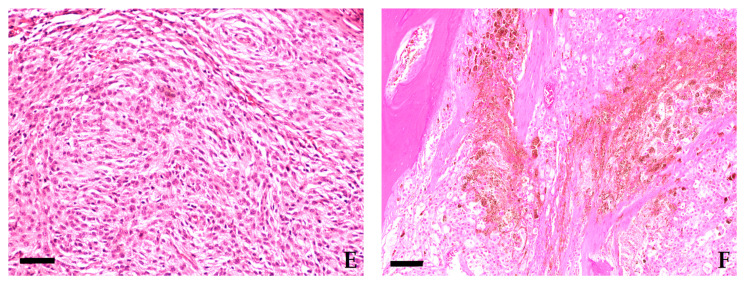
Histological pictures of normal and neoplastic canine toes. (**A**) Normal longitudinal cut through phalanx I of the toe of a 6-year-old Kerry blue Terrier with inflammation in the paw pad (Hematoxylin-Eosin (HE), bar = 1000 µm); (**B**) subungual keratoacanthoma of a 10-year-old English Setter (HE, bar = 250 µm); (**C**) squamous cell carcinoma of a 12-year-old giant Schnauzer (HE, bar = 100 µm); (**D**) apocrine Carcinoma of a 9-year-old Mongrel (HE, bar = 100 µm); (**E**) soft tissue sarcoma of a 9-year-old Dalmatian (HE, bar = 200 µm); (**F**) acral malignant melanoma of a 9-year-old Labradoodle (HE, bar = 200 µm).

Non-neoplastic lesions of the digits/claws (group 1; *n* = 1246) included inflammation (*n* = 1149, 92%; Figure 3A and Figure 4A), cysts originating from the nail bed epithelium or hair follicles (*n* = 61, 5%), epidermal hyperplasia and dyskeratosis (*n* = 18, 1%), or degenerative arthropathy (*n* = 1, 0.1%). The remaining 17 cases (1%) had no pathological alteration in the submitted material. The signalment of the dogs in group 1 is presented in Table 1.

Tumor-like lesions (group 2) were identified in 138 samples (5%), which were divided into focal adnexal dysplasia (n = 124, 90%) and polyps (n = 14, 10%). The signalment of the affected dogs is presented in Table 2.

Digital neoplasms were diagnosed in 1528 cases (group 3). The median age of the dogs in group 3 was 10 years of age (1–17 years; unknown: 149), which was significantly older than both group 1 (7 years of age (0.1–17); unknown: 135; *p* ≤ 0.0001) and group 2 (9 years of age (1–17); unknown: 12; *p* = 0.001).

Digital neoplasms included 335 benign tumors (22%, Table 3): subungual keratoacanthoma (*n* = 217 (Figure 3B and Figure 4B)), histiocytoma (*n* = 36), plasmacytoma (*n* = 35), papilloma (*n* = 16), melanocytoma (*n* = 12), sebaceous gland tumor (*n* = 9), lipoma (*n* = 6), chondroma (*n* = 2), and osteoma (*n* = 2).

Digital malignant neoplasms were identified in 1193 samples (78%, Table 4). This included 758 squamous cell carcinomas (Figure 3C and Figure 4C), 196 melanomas (Figure 3D and Figure 4F), 76 soft tissue sarcomas (Figure 3E and Figure 4E), 52 mast cell tumors (Figure 3F), 37 non-specified sarcomas, 29 anaplastic neoplasms, 24 (apocrine) carcinomas (Figure 4D), 20 malignant bone tumors, and 1 histiocytic sarcoma.

**Figure 5:**
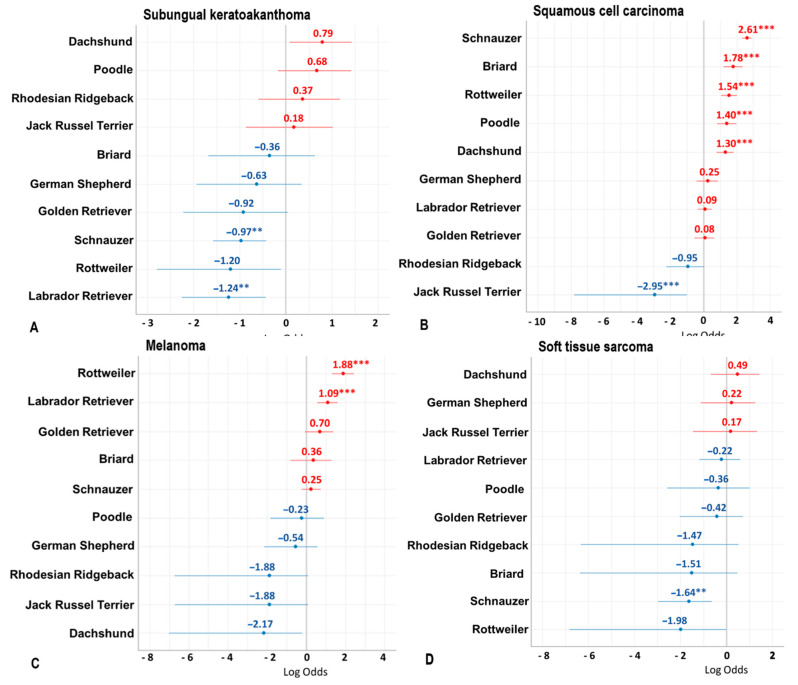
Forest plot of the log odds ratios of subungual keratoacanthoma (**A**), squamous cell carcinoma (**B**), melanomas (**C**), and soft tissue sarcomas (**D**) in the ten most common breeds of the study population (>50 dogs per breed). Positive log odds ratios are depicted in red, negative ones in blue. ** *p* ≤ 0.01; *** *p* ≤ 0.001.

Statistical calculation of breed predisposition or resistance was done for neoplasms and inflammation of the digit with a statistically sufficient number of cases per breed (n > 50):

Schnauzers (log odds ratio (OR) = −0.97) and Labrador Retrievers (log OR = −1.24) had a significantly (*p* ≤ 0.01) lower risk for the development of subungual keratoacanthomas when compared to Mongrels (Figure 5A).

When assessing squamous cell carcinoma, there was a significant (*p* ≤ 0.001) breed predisposition in Schnauzers (log OR = 2.61), Briard (log OR = 1.78), Rottweilers (log OR = 1.54), Poodles (log OR = 1.40), and Dachshunds (log OR = 1.30). In contrast, Jack Russell Terriers (log OR = −2.95) were significantly (*p* ≤ 0.001) less affected (Figure 5B). Interestingly enough, six cases of SCC were in dogs younger than 4 years of age.

Melanocytic neoplasms were detected in 210 cases, most of them with an aggressive behavior and associated bone invasion. In 12 dogs, the pigmented neoplasm was classified as cutaneous melanocytomas of the toe. Digital malignant melanoma was significantly (*p* ≤ 0.001) more common in the Rottweiler (log OR = 1.88) and Labrador Retriever (log OR = 1.09) breeds compared to Mongrels. In contrast, Dachshunds (log OR = −2.17), Jack Russell Terriers (log OR = −1.88), and Rhodesian Ridgebacks (log OR = −1.88) were rarely affected (Figure 5C).

Soft tissue sarcomas (STS) were graded according to McSporran (2009), classified as grade 1 (60.5%), grade 2 (36.6%), or grade 3 (2.9%). This tumor did not seem to show any breed predisposition. However, Schnauzers were significantly (*p* ≤ 0.001) less affected (log OR = −1.64) than Mongrels (Figure 5D).

Given the small number of bone tumors, anaplastic tumors and other carcinomas, or sarcomas, a valid statistical analysis could not be performed in these samples.

Mast cell tumors were commonly diagnosed in Boxers (8/52) and Retrievers (11/52). According to Patnaik et al. (1984), most digital mast cell tumors were grade II (72.2%), while only 14.8% and 13% were classified as grade I and III, respectively.

Inflammation of the digits was significantly (*p* ≤ 0.01) more common in Rhodesian Ridgebacks (log OR = 1.06) than in Mongrels. Schnauzers (log OR = −1.71; *p* ≤ 0.001), Rottweilers (log OR = −1.29; *p* ≤ 0.001), Briards (log OR = −0.95; *p* ≤ 0.01), Poodles (log OR = −0.8; *p* ≤ 0.05), and Dachshunds (log OR = −0.66; *p* ≤ 0.05) showed statistically a lower risk for developing digital inflammation.

## 4. Discussion

Currently, this study represents the largest collection of canine digital lesions (n = 2912) within the existing literature. The incidence of the various lesions described herein are largely in concordance with previous studies [1,3,4] and included a wide age range (1 month to 17 years). As expected, dogs with acral neoplasms were significantly older than those with tumor-like or non-neoplastic changes. This correlates with Wobeser et al. [1], who confirmed a younger age of animals with acral inflammation vs. those with a neoplasia.

Non-neoplastic lesions (92% inflammation, 5% cysts) were identified in 42.7% of all digits, which is higher than in the previous literature (19% Marino et al. [3]; 33% Wobeser et al. [1]). Wobeser et al. [1] found inflammatory lesions of the digits only in 27% of all examined cases, identifying the German Shepherd breed as a predisposed breed. This breed predisposition could not be confirmed by the present study. In contrast, a statistically higher risk of developing inflammatory lesions of the digits was only observed in Rhodesian Ridgebacks.

Tumor-like lesions (composed of focal adnexal dysplasia and polyps) were diagnosed in only 5% of the dogs in our study. Interestingly enough, Labrador Retrievers (19/142), Airedale Terriers (10/142), and American Staffordshire Terriers (AST, 8/142) were the most prevalent pure breeds. Unfortunately, given the small number of affected Aire-dale Terriers and ASTs within the population, breed predisposition statistical analyses were not performed. Nonetheless, none of the breeds were predominately apparent in either group 1 nor 3. Thus, a predisposition of Airedale Terrier as well as of AST for focal adnexal dysplasia of the toe may be suspectable.

In accordance with the literature, neoplastic processes were identified in 52% of all examined amputated canine digits (65.5% Gruber-Beckmann et al. [4]; 73% Wobeser et al. [1]; 81% Marino et al. [3]). Of these, in descending prevalence, squamous cell carcinomas, melanomas, and soft tissue sarcomas were the most frequently diagnosed malignant neoplasms [1,4,19]. Interestingly enough, the results of the current study differ somewhat, which may be explained by the different regional variation of the dog populations at this time. Supporting this hypothesis, according to an online comparison portal for dog insurance in Germany (Check24) and the registration portal Tasso (www.tasso.net, accessed on 28 April 2020), mixed breeds, Labrador Retrievers, German Shepherds, French Bulldogs, Chihuahuas, Australian Shepherds, Jack Russell Terriers, and Yorkshire Terriers were the most frequently reported dog breeds in 2016–2019. In contrast, Bernese Mountain Dogs are more common in Switzerland [20,21]. Since fighting dog breeds like Staffordshire and Bull Terriers have been banned in Germany for years, their numbers in Germany are low in comparison to older studies from other countries like Canada [1] or the UK [22]. With this in mind and with the exceptional popularity of Schnauzers in Germany, the present study population seems to be a good representation of the current dog population. Schnauzers are particularly common in Germany due to their Germanic origin [23], thus their popularity. For this reason, a shift of our data towards the Schnauzer and SCC is to be expected, given that the majority of the samples are from Germany and that this breed is predisposed to the development of digital SCC.

The distinction between subungual keratoacanthoma from well-differentiated SCC can be somewhat challenging depending on the pathologist´s experience [24]. However, in the present study, subungual keratoacanthoma was significantly less common in Schnauzers and Labrador Retrievers than in Mongrels. This could be due to the relatively high predisposition of Schnauzers and Labrador Retrievers to develop SCC and melanoma, respectively. The low prevalence of Schnauzers developing subungual keratoacanthoma may suggest a de novo development of digital SCC rather than a malignant transformation of pre-existing subungual keratoacanthomas into SCC. On the other hand, Dachshunds and Poodles, have a high risk of developing both SCC and subungual keratoacanthoma. In conclusion, this suggests different pathogenetic pathways for the development of digital SCC, depending on the breed.

The current study confirmed the already well-known breed predisposition for the development of SCC in Schnauzers, Briards, Rottweilers, Poodles, and Dachshunds [5,6,19]. However, the prevalence of SCC was higher in our study than in previous literature [1,2,3]. As already mentioned, this is probably caused by the high proportion of Schnauzers included in our study cohort. Karyadi et al. [25] found a copy number variant at the KITLG locus, likely responsible for high the risk of subungual SCC for black Poodles. Hypothetically, if this copy number variation is also responsible for other predisposed breeds (e.g., Schnauzer) this must be investigated in further studies. Additionally, a previous study demonstrated that the digital SCC of dark-haired breeds had more histologically malignant features than their light-haired counterparts [26]. Nevertheless, this represents the first study identifying Jack Russell Terriers to have a significantly lower risk of developing digital SCC. This seems plausible since Jack Russell Terriers usually have white paws. This particular white color is because of “white spotting” or “extreme white spotting” caused by a well described variant at the S-locus [27]. This variant stops the migration of melanocytes and leaves certain areas of the skin and hair devoid of pigment. The Jack Russell Terrier breed-standard demands “White or predominantly white with tan, lemon or black markings, or any combination of these colors. The color preferably confined to the head and/or root of tail, but a little body color is acceptable.” [28]. Therefore, Jack Russell Terriers are expected to be genetically homozygous for the “white spotting” or “extreme white allele” “S” on the S-locus, exhibiting white hair, colorless claws and pink skin on the feet in nearly every case. Interestingly enough, Tompkins et al. [29] differentiated the sites of SCC in the skin of the nail bed and other sites. They found out that SCC were most commonly develops in the nail bed of Rottweilers and Golden Retrievers, while the cutaneous SCC was most frequently seen in Jack Russell Terriers. In the present study, Jack Russell Terriers were seen to have even a decreased incidence of SCC of the toes. This difference suggests that the anatomical site may be an additional important factor in oncogenesis. In humans, SCC of the nail beds are reported infrequently and associations with papillomavirus, immunosuppression, tobacco use, dominant handedness, age, toxin or radiation use as well as trauma are discussed [30]. Similar to dogs, the incidence of SCC in humans shows marked variation in its distribution, suggesting that personal habits, environmental exposures, infections, and ethics play all different roles in the etiology of SCCs in various anatomical sites [31]. Consequently, further studies are necessary to determine the etiological factors of SCC in different anatomical locations in dogs.

Similar to Grüntzig et al. [6], SCC were less common in females than males. In the total study population, intact males were twice as common as neutered males, while the ratio of neutered to non-neutered bitches was about the same. These relationships were roughly reflected in all tumors. Therefore, a conclusion concerning an influence of castration in the development of these tumors could not be drawn.

In the present study, Rottweilers and Labrador Retrievers had a significantly higher risk for melanoma than Mongrels. This is similar to the available literature [11], in which both breeds were predisposed for the development of melanoma. Interestingly, these two breeds share a high proportion of DNA sequence and seem to be closely related [32], so an underlying, common, genetic factor can be hypothesized. In the literature, the most common genes related to the development of melanomas in dogs are dependent on location: PTEN (mucosal) [11,33,34], TP53 (mucosal, skin) [33,34], KIT (skin) [33], BRAF (skin) [35] as well as NRAS and KRAS (mucosal, skin, digit) [11,33,34]. Nevertheless, although Dachshunds have a higher risk for oral and dermal melanomas [9], in the present study, this breed had the lowest risk of developing digital melanocytic neoplasms. Furthermore, the Rhodesian Ridgeback is predisposed to cutaneous melanocytoma [36] but rarely develops digital melanoma when compared to Mongrels. Thus, a breed predisposition correlated to a specific tumor site seems to be likely in these two breeds, as previously discussed for cutaneous/digital SCC in Jack Russell Terriers. Further studies are necessary to clarify the underlying pathogenetic mechanism.

Mast cell tumors (MCT) were common in Boxers, Retrievers, and French Bulldogs, similar to the description by Pierini et al. [37], Kok et al. [38], and Lapsley and Selmic [19]. American Pitbulls, described as a predisposed breed by Pierini et al. [37], did not appear in our list, most likely because of their limited ownership in several countries. In general, cutaneous MCT were, according to Patnaik et al. [39], most commonly classified as grade II [38,40]. However, currently, there is no grading regarding digital mast cell tumors. Nonetheless, in the present study, most MCT belonged to grade II [39], a low grade [41]. However, given the location, resection of the mass with wide margins is challenging [40], with digital amputation often recommended as the treatment of choice.

Soft tissue sarcoma (STS) arise in 60% of the cases on the limbs, although a breed predisposition was not identified [42], similar to the present study. The fact that Schnauzers seem to have a lower predisposition to the development of STS may result from its extremely high proportion of SCC instead, as discussed above.

Osteosarcomas are often described in large breeds, such as Great Dane, Leonberger, Rottweiler, Irish Wolfhound, among others [43,44]. However, in our study, only few of these breeds were included in samples shown to be developing osteosarcoma, with the exception of Rottweilers. This is likely due to the examined anatomical location of tge “toe” in the current study, in which osteosarcomas are not frequently seen.

There was a number of non-specified sarcomas, carcinomas, and anaplastic neoplasms that did not reveal any special information regarding breed, age, or sex predispositions of the affected animals. However, it should be noted that eight dogs with digital malignant neoplasms were younger than 4 years of age.

In cats, “digit–lung syndrome” is described in cases of primary pulmonary neoplasms with metastasis in the digits [45]. On the one hand, This most likely results from the angioinvasive properties of these neoplasms [45] and on the other hand, from the high digital blood flow to facilitate heat loss in cats [46]. Due to the lack of anamnestic data, the previous study enables no statement regarding this syndrome in dogs.

In summary, this is the first study identifying breeds with high as well as low risks for the development of certain digital neoplasia. It should be noted that when specifying breed predispositions, the total dog population and the breed representation within that group must be taken into account. Additionally, in order to perform statistical analyses, a minimum number of cases is necessary to produce reliable results. In the present study, although a large number of cases was included, some discrete breed predispositions or resistances may not have been detected due to only few cases in that specific breed. In the future, national and international tumor registries (e.g., https://www.givcs.org/, accessed on 28 April 2020) could be helpful for such analyses [47] by providing even a larger caseload.

Underlying causes for certain breed predispositions for neoplasia development are largely unknown, but the specific immune response could play a role. Villaescusa et al. [48] reported a significantly different CD4/CD8 ratio between Labrador Retrievers and German Shepherds living in the same environment. Immunotherapy may play an upcoming role in the therapy of canine melanomas [49] and osteosarcomas [50]. Various mutations in canine MM [51], e.g., RAS mutation in canine digital melanoma [33], and copy number variations of the KITLG Locus in digital SCC [25,52] have been identified to play a role in oncogenesis.

## 5. Conclusions

The present study is, to our knowledge, the first to identify low-risk breeds and the development of certain neoplastic conditions on the canine digit. These relative resistances were suggested for Jack Russell Terriers (SCC of the toe) as well as for the Dachshunds and Rhodesian Ridgebacks (acral MM). This represents a milestone in order to identify further genetic and immunological factors that may predispose certain breeds to “tumor resistance” against specific digital neoplasia and for future oncotherapy.

## Figures and Tables

**Table 1:** Signalment of the 1246 dogs with non-neoplastic lesions (group 1).

Diagnosis	Breeds	Median Age (Range) in Years	Sex
Inflammation (*n* = 1149)	291 Mongrels, 76 Labrador Retrievers, 51 Schnauzers, 47 Golden Retrievers, 38 German Shepherds, 37 Rhodesian Ridgebacks, 28 JRT, 22 Belgian Shepherds, 22 Boxers, 21 Dachshunds, 20 French Bulldogs, 20 Spaniels, 18 Great Danes, 17 Dobermanns, 16 Rottweilers, 15 BMDs, 15 WHWTs, 14 Bull Terriers, 14 Pugs, 14 Poodles, 13 Briards, 12 Beagles, 12 Setters, 11 Collies, 11 Irish Wolfhounds, 10 YTs, 8 Australian Shepherds, 8 Tibet Terriers, 7 Old German Shepherds, 7 ASTs, 7 English Bulldogs, 7 Fox Terriers, 7 Greyhounds, 7 Shih Tzus, 6 Bearded Collies, 6 Border Collies, 6 German Wire-haired Pointers, 6 Miniature Schnauzers, 5 Dalmatians, 5 Huskies, 5 Maltese, 5 White Swiss Shepherds, 4 Chihuahuas, 4 German Short-haired Pointers, 4 FCRs, 4 Spanish Greyhounds, 4 Hovawarts, 4 Olde English Bulldogs, 4 Parson Russell Terriers, 4 Russian Black Terriers, 4 Shetland Sheepdogs (Sheltie), 4 Weimaraners, 4 Whippets, 3 Airedale Terriers, 3 Akitas, 3 American Bulldogs, 3 Beaucerons, 3 Cairn Terriers, 3 Chow Chows, 3 Dogos Argentinos, 3 Irish Terriers, 3 Leonbergers, 3 Magyar Viszlas, 3 Podencos, 3 SBTs, 102 dogs from 58 other breeds	7 (0.1–17) 121 U	221 F, 206 FS, 415 M, 221 MC, 86 U
Cyst (*n* = 61)	19 Mongrels, 3 Chihuahuas, 3 Great Danes, 3 Pugs, 33 dogs from 28 other breeds	9 (2–14) 9 U	13 F, 18 FS, 15 M, 9 MC, 6 U
Hyperplasia/dyskeratosis (*n* = 18)	6 Mongrels, 3 Labrador Retrievers, 9 dogs from 9 other breeds	6 (1–14) 1 U	2 F, 3 FS, 8 M, 4 MC, 1 U
Normal toe/claw (*n* = 17)	4 Schnauzers, 3 Mongrels, 2 Golden Retrievers, 8 dogs from 8 other breeds	8 (2–11) 2 U	5 FS, 6 M, 3 MC, 3 U
Arthropathia (*n* = 1)	1 Dobermann	1 U	1 F

**Table 2:** Signalment of 138 dogs with tumor-like lesions (group 2).

Diagnosis	Breeds	Median Age (Range) in Years	Sex
Focal adnexal dysplasia (*n* = 124)	25 Mongrels, 19 Labrador Retrievers, 10 Airedale Terriers, 8 ASTs, 4 Old German Herding Dogs, 4 JRTs, 4 Greyhounds, 3 Alaska Malamutes, 3 American Bulldogs, 3 Boxers, 3 Golden Retrievers, 3 Huskies, 3 Magyar Vizslas, 32 dogs from 26 other breeds	9 (1–16) 10 U	15 F, 25 FS, 43 M, 32 MC, 9 U
Polyp (*n* = 14)	5 Mongrel, 9 dogs from 9 other breeds	9 (2–14) 9 U	2 F, 1 FS, 3 M, 6 MC, 2 U

**Table 3:** Signalment of the 335 dogs with benign tumors (group 3a).

Diagnosis	Breeds	Median Age (Range) in Years	Sex
Subungual keratoacanthoma (*n* = 217)	55 Mongrels, 14 Schnauzers, 12 Dachshunds, 8 Poodles, 6 BMDs, 6 FCRs, 6 Rhodesian Ridgebacks, 6 YTs, 5 JRTs, 5 Labrador Retrievers, 5 Spaniels, 4 Beagles, 4 Hovawarts, 4 Setters, 3 Beaucerons, 3 Bolonki Zwetnas, 3 Border Collies, 3 Briards, 3 German Shepherds, 3 Dobermanns, 3 English Pointers, 3 Golden Retrievers, 3 Greyhounds, 3 Shetland Sheepdogs (Sheltie), 3 Shih Tzus, 44 dogs from 38 other breeds	10 (1–16) 21 U	42 F, 48 FS, 68 M, 49 MC, 10 U
Histiocytoma (*n* = 36)	12 Mongrels, 7 French Bulldogs, 4 JRTs, 2 Boxers, 2 Magyar Vizslas, 9 dogs from 9 other breeds	5 (1–12) 4 U	7 F, 5 FS, 11 M, 11 MC, 2 U
Plasma cell tumor (*n* = 35)	6 Mongrels, 4 JRTs, 3 French Bulldogs, 3 Labrador Retrievers, 3 Schnauzers, 3 WHWTs, 2 YTs, 11 dogs from 11 other breeds	10 (3–16) 3 U	6 F, 6 FS, 14 M, 7 MC, 2 U
Papilloma (*n* = 16)	6 Mongrels, 2 Pinschers, 2 JRTs, 2 Huskies, 4 dogs from 4 other breeds	8 (2–14) 3 U	3 F, 5 FS, 4 M, 2 MC, 2 U
Melanocytoma (*n* = 12)	3 Labrador Retrievers, 2 Briards, 7 dogs from 7 other breeds	10 (5–12) 4 U	1 F, 4 FS, 3 M, 4 MC
Tumor of the sebaceous glands (*n* = 9)	3 Mongrels, 2 Labrador Retrievers, 4 dogs from 4 other breeds	11 (11–14)	4 FS, 3 M, 2 MC
Lipoma (*n* = 6)	4 Mongrels, 2 dogs from 2 other breeds	10 (4–12) 1 U	2 FS, 2 MC, 2 U
Benign bone tumor (*n* = 4)	1 French Bulldog, 1 Great Dane, 1 Australian Shepherd, 1 English Bulldog	6 (3–9)	1 FS, 1 M, 1 MC, 1 U

**Table 4:** Signalment of the 1193 dogs with malignant tumors (group 3b).

Diagnosis	Breeds	Median Age (Range) in Years	Sex
Squamous cell carcinoma (*n* = 758)	298 Schnauzers, 98 Mongrels, 40 Rottweilers, 33 Labrador Retrievers, 28 Briards, 28 Dachshunds, 27 FCRs, 22 Poodles, 20 Setters, 15 Golden Retrievers, 13 German Shepherds, 11 Miniature Schnauzers, 8 BMD, 8 Hovawarts, 7 Russian Black Terriers, 7 Spaniels, 6 Airedale Terriers, 6 German Short-haired Pointers, 4 Australian Shepherds, 3 Beaucerons, 3 Boxers, 3 Kerry Blue Terriers, 3 Leonbergers, 3 Rhodesian Ridgebacks, 3 WHWTs, 3 YTs, 58 dogs from 42 other breeds	10 (3–16) 72 U	155 F, 165 FS, 268 M, 111 MC, 59 U
Malignant melanoma (*n* = 196)	39 Mongrels, 31 Labrador Retrievers, 31 Schnauzers, 25 Rottweilers, 9 Golden Retrievers, 6 Spaniels, 4 Briards, 4 Dobermanns, 3 Russian Black Terriers, 3 Scottish Terriers, 41 dogs from 31 other breeds	10 (2–16) 20 U	47 F, 30 FS, 75 M, 29 MC, 15 U
Soft tissue sarcoma (*n* = 76)	21 Mongrels, 5 Labrador Retrievers, 4 Dachshunds, 3 German Shepherds, 43 dogs from 33 other breeds	11 (5–17) 4 U	16 F, 19 FS, 29 M, 8 MC, 4 U
Mast cell tumor (*n* = 52)	8 Boxers, 6 Mongrels, 6 Labrador Retrievers, 5 Golden Retrievers, 4 BMDs, 4 French Bulldogs, 19 dogs from 19 other breeds	8 (2–16) 8 U	13 F, 8 FS, 18 M, 9 MC, 4 U
Other sarcoma (*n* = 37)	10 Mongrels, 3 Labrador Retrievers, 3 German Shepherds, 3 Australian Shepherds, 2 Beagles, 16 dogs from 16 other breeds	10 (5–16) 1 U	10 F, 12 FS, 8 M, 5 MC, 2 U
Anaplastic tumor (*n* = 29)	9 Mongrels, 4 Golden Retrievers, 3 Labrador Retrievers, 3 Schnauzers, 10 dogs from 10 other breeds	10 (2–17) 6 U	9 F, 5 FS, 10 M, 4 MC, 1 U
(Apocrine) Carcinoma (*n* = 24)	9 Mongrels, 15 dogs from 15 other breeds	11 (8–14) 4 U	3 F, 6 FS, 7 M, 7 MC, 1 U
Malignant bone tumor (*n* = 20)	6 Mongrels, 2 German Shepherds, 2 Labrador Retrievers, 2 Italian Cani Corsi, 8 dogs from 8 other breeds	9 (3–13) 4 U	6 F, 4 FS, 5 M, 4 MC, 1 U
Histiocytic sarcoma (*n* = 1)	1 Mongrel	9	1 MC

## Data Availability

Data available upon request due to restrictions e.g., privacy or ethical.

## Abbreviations

AST = American Staffordshire Terrier, BMD = Bernese Mountain Dog, F = female, FS = female spayed, FCR = Flat Coated Retriever, JRT = Jack Russell Terrier, M = male, MC = male castrated, MM = malignant melanoma, SBT = Staffordshire Bull Terrier, SCC = squamous cell carcinoma, STS = soft tissue sarcoma, WHWT = West Highland White Terrier, U = unknown, YT = Yorkshire Terrier.
